# Digital Microfluidic Mixing via Reciprocating Motions of Droplets Driven by Contact Charge Electrophoresis

**DOI:** 10.3390/mi13040593

**Published:** 2022-04-10

**Authors:** Jaewook Kim, Taeyung Kim, Inseo Ji, Jiwoo Hong

**Affiliations:** School of Mechanical Engineering, Soongsil University, 369 Sangdo-Ro, Dongjak-Gu, Seoul 06978, Korea; jaewuk1024@gmail.com (J.K.); rlaxodudsla@gmail.com (T.K.); inseotropy@gmail.com (I.J.)

**Keywords:** digital microfluidics, lab-on-a-chip, contact charge electrophoresis, droplet mixing, droplet reciprocating motions

## Abstract

Contact charge electrophoresis (CCEP) is an electrically controllable manipulation technique of conductive droplets and particles by charging and discharging when in contact with the electrode. Given its straightforward operation mechanism, low cost, and ease of system construction, it has gained traction as a versatile and potential strategy for the realistic establishment of lab-on-a-chip (LOC) in various engineering applications. We present a CCEP-based digital microfluidics (DMF) platform with two parallel electrode modules comprising assembled conventional pin header sockets, allowing for efficient mixing through horizontal and vertical shaking via droplet reciprocating motions. The temporal chromic change caused by the chemical reaction between the pH indicator and base solutions within the shaking droplets is quantitatively analyzed under various CCEP actuation conditions to evaluate the mixing performance in shaking droplets by vertical and horizontal reciprocating motions on the DMF platform. Furthermore, mixing flow patterns within shaking droplets are successfully visualized by a high-speed camera system. The suggested techniques can mix samples and reagents rapidly and efficiently in droplet-based microreactors for DMF applications, such as biochemical analysis and medical diagnostics.

## 1. Introduction

Digital microfluidics (DMF) is a sophisticated and versatile technology for manipulating microdroplets on demand which allows for the creation of lab-on-a-chip (LOC) platforms by using an array of independently controlled electrodes and programmable electrical signals [1,2,3,4]. The DMF platform enables the creation, transportation, integration, fusion, and reaction of droplets carrying biological (or chemical) samples (or reagents) with volumes ranging from microliter to nanoliter without a mechanical-fluidic control apparatus [3,4]. Given these advantages, the DMF platform has been widely used in various biomedical and biochemical sectors, including digital polymerase chain reaction assays, cell culture, and drug screening [3,4].

Electrowetting-on-dielectric (EWOD) has long been acknowledged as the most common technology for building the DMF platform and a potential candidate for commercialization in industrial areas [5,6,7,8]. Here, EWOD refers to a technique for manipulating droplets in which the contact angle (or contact line) of a conducting discrete liquid placed on a dielectric-covered electrode array can be electrically controlled [9,10]. EWOD enables precise and rapid handling of microdroplets while costing less power. In addition, its effortless individual addressability of electrodes allows for the simultaneous handling of microdroplets containing different samples (or reagents). Despite these technological advantages, it has the disadvantage of being difficult to operate when dealing with non-liquid objects, such as gels or solids [11,12]. For example, if two droplets carrying different reactants meet and chemically react to gelify or solidify on the EWOD chip, it loses its ability to manipulate [12]. Furthermore, complicated, time-consuming, and costly lithographic microfabrication processes are required to fabricate and extend the electrode array [13].

Contact charge electrophoresis (CCEP) has been gained traction as an easy-to-use technology capable of competing with EWOD in the DMF research realm [12,14,15,16]. CCEP allows for the electrically controlled direction of conductive droplets and particles by charging and discharging when in contact with electrodes [16]. Compared with EWOD, CCEP is capable of directing gel or solid particles in addition to droplets, thereby minimizing the contact area between the objects and the solid electrodes [12]. Additionally, an electrode array can be readily constructed without using lithographic microfabrication procedures by assembling pin header sockets that are routinely used in conventional electronics. For example, Im and his colleagues built the first CCEP-based DMF platforms with electrode arrays made of ordinary pin header sockets with a 2.54 mm pitch, enabling 2D droplet manipulation [12]. Review papers provide a concise summary of the fundamentals and DMF applications of CCEP-based particle and droplet manipulation [14,15,16].

Despite the passionate extensive efforts of researchers focusing on DMF-related research based on the CCEP, technical issues remain to be resolved. Compared with EWOD technology, the CCEP technology’s droplet manipulation capabilities are primarily limited to transporting and merging. A few papers, to the best of our knowledge, have reported simple mixing performance via horizontal reciprocation and the merging of two droplets via CCEP manipulation on a two-dimensional DMF platform to broaden the CCEP droplet handling function [12,17]. However, the time required to completely mix different reagents (i.e., phosphate-buffered saline and calcium chloride) in horizontally shaking droplets employing CCEP actuation took up two minutes or longer in that research [12].

**Figure 1:**
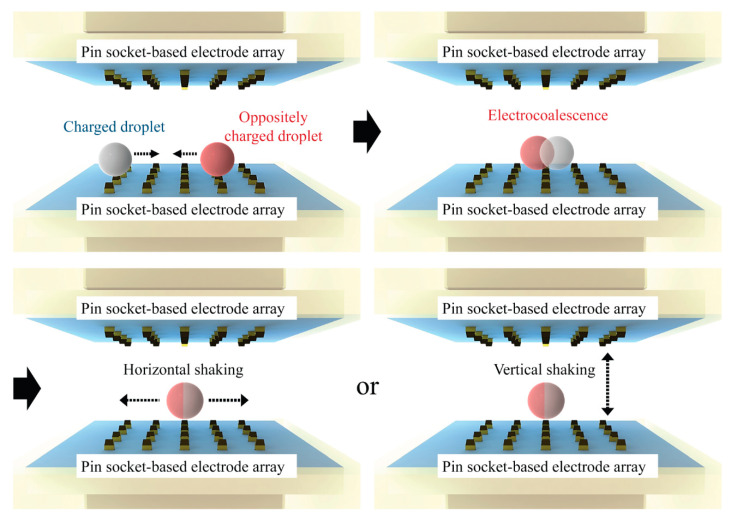
Conceptual diagram of the proposed digital microfluidic mixing via the reciprocating motion of droplets driven by contact charge electrophoresis.

For effective active mixing, a three-dimensional (3D) DMF platform with two parallel electrode modules comprising assembled pin header sockets (2 × 5 pins with 2 mm pitch) is designed in this study, as illustrated in Figure 1. Droplets can therefore reciprocate horizontally and vertically in response to the CCEP actuation on the platform, causing horizontal and vertical droplet shaking, respectively. In this study, pin header sockets with a narrow pitch (2 mm) are used instead of the previously used sockets with a 2.54 mm pitch [12,18,19]. Consequently, the same electric field can be generated at a lower voltage and a more compact DMF platform than in previous studies [12]. A temporal color change experiment by the chemical reaction between sodium hydroxide aqueous solution and phenolphthalein (PHP) indicator solution is performed to compare and evaluate the mixing performance in shaking droplets by vertical and horizontal reciprocating droplet motions on the CCEP-based DMF platform.

## 2. Materials and Methods

**Figure 2:**
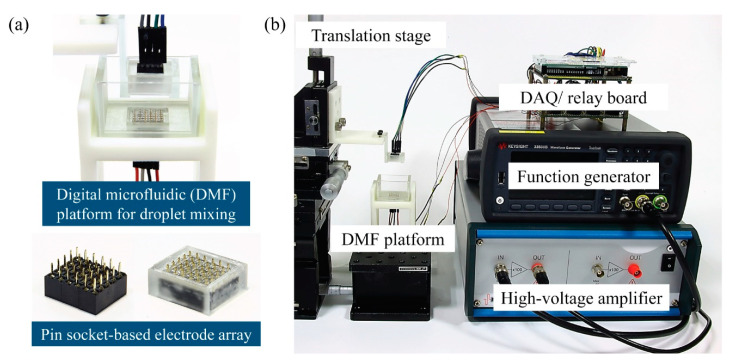
(**a**) Schematic of the digital microfluidic (DMF) platform for droplet mixing using reciprocating droplet motion driven by contact charge electrophoresis (CCEP). The electrode system of the DMF platform comprises a small commercial pin socket and a 3D-printed enclosure. (**b**) Entire experimental setup for DMF mixing with CCEP actuations.

The schematic of the DMF platform for droplet mixing with reciprocating droplet motion driven by CCEP is shown in Figure 2, as well as the entire experimental setup for steering droplet motion using CCEP actuation. The electrode module of the DMF platform comprises a pin-typed electrode array assembled by combining three (6 × 5) pin header sockets (2 × 5 pins with 2 mm pitch) that are commonly used in conventional electronics (bottom in Figure 2a). In this study, pin header sockets with a narrow pitch (2 mm) are used instead of sockets with a 2.54 mm pitch that were previously used [12,18,19]. As a result, the same electric field can be created at a lower voltage and a more compact DMF platform can be built than in previous studies [12]. The frames with holes that are nearly the same size as the pin of electrodes are manufactured with a digital light processing (DLP) 3D printer (Asiga MAX UV, Asiga, Alexandria, Australia) and printable resin (PlasClear V2, Asiga) to hold the pin header sockets and prevent liquid leakage by exposing only the pin portion of the sockets. Following insertion of the sockets into the 3D-printed frame, the gap between the pin-typed electrodes is filled with polydimethylsiloxane (PDMS), creating a smooth surface and preventing dielectric oil medium leakage. Notably, the amount of PDMS used is adjusted so that only the tip of the electrode pin is exposed. In this instance, the PDMS precursor mixture (Sylgard 184, Dow Corning, Midland, MI, USA) is used at a 7:1 weight ratio of base to curing agent. The electrode module filled with the PDMS is cured for two hours at 100 °C in an oven. Then, the surface of the electrode module is hydrophobically coated with a Teflon solution containing 0.2% (v/v) Teflon AF1600 (DuPont), dissolved in FC-40 solvent (Sigma Aldrich, St. Louis, MO, USA), and baked at 110 °C for 5 min. After washing with isopropyl alcohol and subsequent air blowing, the Teflon coating remained only on the PDMS surface. Thus, the pinning of the droplets on solid surfaces can be prevented during CCEP actuation. Except for the pinning problem, it is found that whether the Teflon coating is treated or not has no effect on the charging process of the droplets or subsequent movement behaviors after charging.

A DMF platform with two parallel electrode modules is constructed, allowing droplets to reciprocate horizontally and vertically in response to the CCEP actuation (top in Figure 2a). The lower electrode module is mounted in a U-shaped chamber created with a DLP 3D printer and printable resin. Thereafter, transparent acrylic plates with a 2 mm thickness are attached to the chamber’s walls to contain a dielectric oil medium and allow front and back view observations. The upper electrode module is linked to the translation stage through a 3D printed supporter. By adopting a translation stage, the vertical distance between the upper and lower electrodes can be manually adjusted between 2 mm and 4 mm in a 1 mm increment.

The PHP reaction is introduced to visualize and quantify the mixing process that occurs during the reciprocating motions of droplets driven by the CCEP actuation because the color of PHP changes from colorless to pink when it reacts with bases. For this, sodium hydroxide (NaOH) aqueous solution (0.4 wt%, Comscience) and PHP indicator solution (ethanol:distilled water:PHP = 79:20:1 in wt%, Taejin Chemical, Seoul, Korea) are used. Two droplets with a fixed volume of 0.2 ± 0.05 μL (equivalent to a diameter of 0.72 ± 0.06 mm) each of sodium hydroxide aqueous solution and PHP indicator solution are gently dispensed into a chamber filled with silicone oil (KF-96L, ShinEtsu, dynamic viscosity of 28.6 mPa·s) used as the dielectric suspending medium. The current DMF system, which employs ordinary header sockets with a 2 mm pitch, enables reliable 3D manipulation of droplets with volumes less than 1.5 μL (equivalent to a diameter of 1.4 mm) without risk of electrolysis. The densities of NaOH aqueous solution, PHP indicator solution, and silicone oil are 1.01 g/cm^3^, 0.78 g/cm^3^, and 0.95 g/cm^3^, respectively.

A data acquisition (DAQ) board based on an open-source microcontroller Arduino (Arduino mega 2560) is employed to apply programmable electrical voltages to each pin-typed electrode. The relays with 10 channels are controlled by an Arduino and transfer signals to a DAQ board. DC electrical signals are generated using a function generator (33500B, Agilent, Santa Clara, CA, USA), which are then amplified by a high-voltage amplifier (A800D, Pendulum, Banino, Poland). The applied voltage ranges from 200 V to 400 V, with increments of 100 V. Our high-voltage amplifier has a maximum input voltage of 4 V. Thus, the maximum applied voltage in this experiment is limited to 400 V. When the relay is activated, the amplified voltage is applied to the pin-typed electrode linked to the channel.

**Figure 3:**
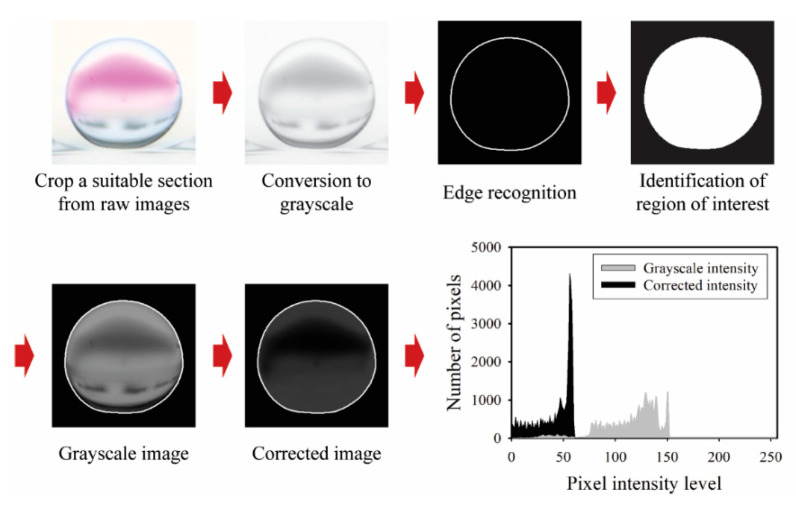
Schematic of the imaging process procedures used to determine the temporal variation in the mixing index within CCEP-actuated shaking droplets.

The droplet motion and mixing process within shaking droplets under CCEP actuation are consecutively recorded at 120 frames per second using a digital camera (EOS 90D, Canon, Tokyo, Japan) equipped with a macro lens (MP-E 65 mm, Canon). Internal flow patterns within the shaking droplets are also successively observed using a high-speed camera (Fastcam Mini UX100, Photron, Tokyo, Japan) fitted with a macro lens at frame rates ranging from 250 to 1000 frames per second. To extract quantitative information from the acquired consecutive images, such as temporal variations of displacement and mixing index of shaking droplets by horizontal and vertical reciprocating motions, digital image processing and data analysis are performed using Adobe Premiere Pro software (v22.0.0, Adobe, Mountain View, CA, USA) and an in-house MATLAB-based program (MATLAB R2021A, MathWorks, Natick, MA, USA). The droplet transport velocity is estimated by extracting information on the change in droplet center displacement over time from successive images acquired. Meanwhile, the mixing index is calculated using the imaging process shown in Figure 3 to extract intensity information on the change in color of shaking droplets during the PHP reaction. The imaging process for calculating the mixing index has the following steps: (1) a suitable section (450 × 450 pixels) is selected and cropped from raw images, (2) the image type is converted from RGB to grayscale, (3) the region of interest (ROI) is identified after edge recognition, (4) images within the ROI are corrected by subtracting the grayscale intensity value from the green intensity value [20], (5) information on the number of pixels versus pixel intensity level from the corrected image is acquired, as shown in the histogram of Figure 3. Note that the monochromic information on images converted to grayscale is employed solely for the purpose of identifying the region of interest (ROI). After determining the ROI, the mixing index is calculated using the color information. The standard deviation of the corrected intensity (*σ*) is calculated using the following formula [20]:

$$\sigma ={\left[\frac{1}{n}\sum_{i=1}^{n}{\left(\frac{I_{i}-{\bar{I}}_{min}}{{\bar{I}}_{max}}\right)}^{2}\right]}^{1/2}.$$

Here, *n* is the number of pixels within the ROI, *I_i_* is the corrected intensity of pixel *i*-th within the ROI, ${\bar{I}}_{min}$ and ${\bar{I}}_{max}$ are the image set average values of the maximum and minimum pixel intensities of each frame, respectively. When mixing is not performed, the standard deviation of the corrected intensity equals one, whereas when full mixing is performed, it should equal zero as all pixels achieve uniform darkest intensity. The level of the mixing index (*M*) is calculated as follows [21]:

$$M\left(\%\right)=\left(1-\frac{\sigma -\sigma_{final}}{\sigma_{final}}\right)\times 100$$

Here, *σ_f_* denotes the standard deviation of each experimental image set’s last frame.

## 3. Results and Discussion

**Figure 4:**
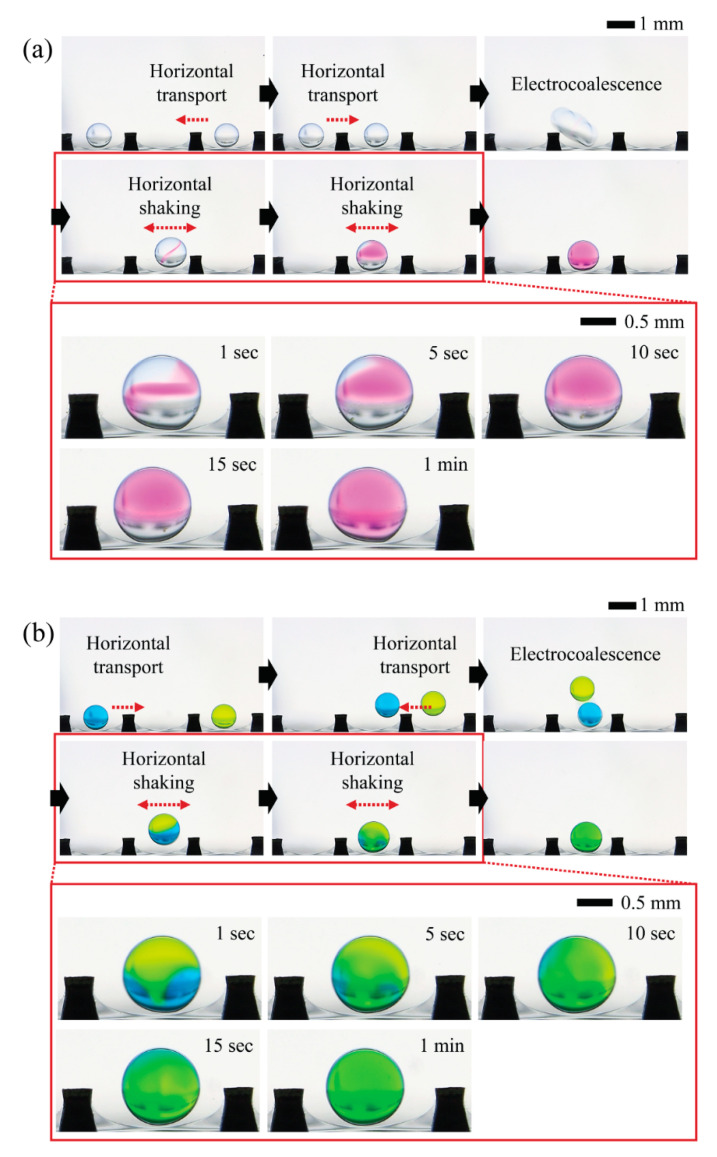
Programmable droplet handling procedure including horizontal transport of two oppositely charged microdroplets containing different chemicals, merging via electrical interaction (i.e., electrocoalescence), and horizontal shaking via horizontal reciprocating motion. (**a**) Mixing between a droplet of PHP indicator solution (left) and a droplet of sodium hydroxide aqueous solution (right) (Video S1 in Supplementary Materials). (**b**) Mixing between a blue dye droplet (left) and a yellow dye droplet (right) (Video S2 in Supplementary Materials). The distance between the pin electrodes is 2 mm in this case, and the applied voltage is 400 V.

To qualitatively show the mixing process within the shaking droplets induced by reciprocating motions of droplets in response to CCEP actuation, we preferentially examine the possibility of programmable droplet handling in the horizontal direction, which includes horizontal transport of two oppositely charged microdroplets containing different chemicals, merging via electrical interaction (i.e., electrocoalescence), and horizontal shaking via horizontal reciprocating motion (Figure 4). When a droplet of NaOH solution comes in contact with the ground electrode (the droplet on the right in the first image of Figure 4a), it becomes negatively charged. Then, by sequentially sending a positive signal to neighboring electrodes, the droplet can be moved horizontally to the left. When a droplet of PHP indicator solution encounters a positively activated electrode (the droplet on the left in the initial image of Figure 4a), the droplet becomes positively charged. By sequentially delivering a ground signal to neighboring electrodes, the droplet can be moved horizontally to the right. When two droplets with opposite charges meet, they are merged electrically through the electrostatic interaction. Finally, the electrocoalesced droplets reciprocate horizontally between the positive and ground electrodes. By monitoring the chromatic change of PHP from colorless to pink, we confirm that mixing proceeds and is completed within roughly one minute during the reciprocating motion of the droplet. Later in this section, quantitative information on mixing performance will be presented and discussed. Similarly, we demonstrate that droplets of different colors can be horizontally transported, merged, and mixed using the same droplet handling procedures, as shown in Figure 4b. 

**Figure 5:**
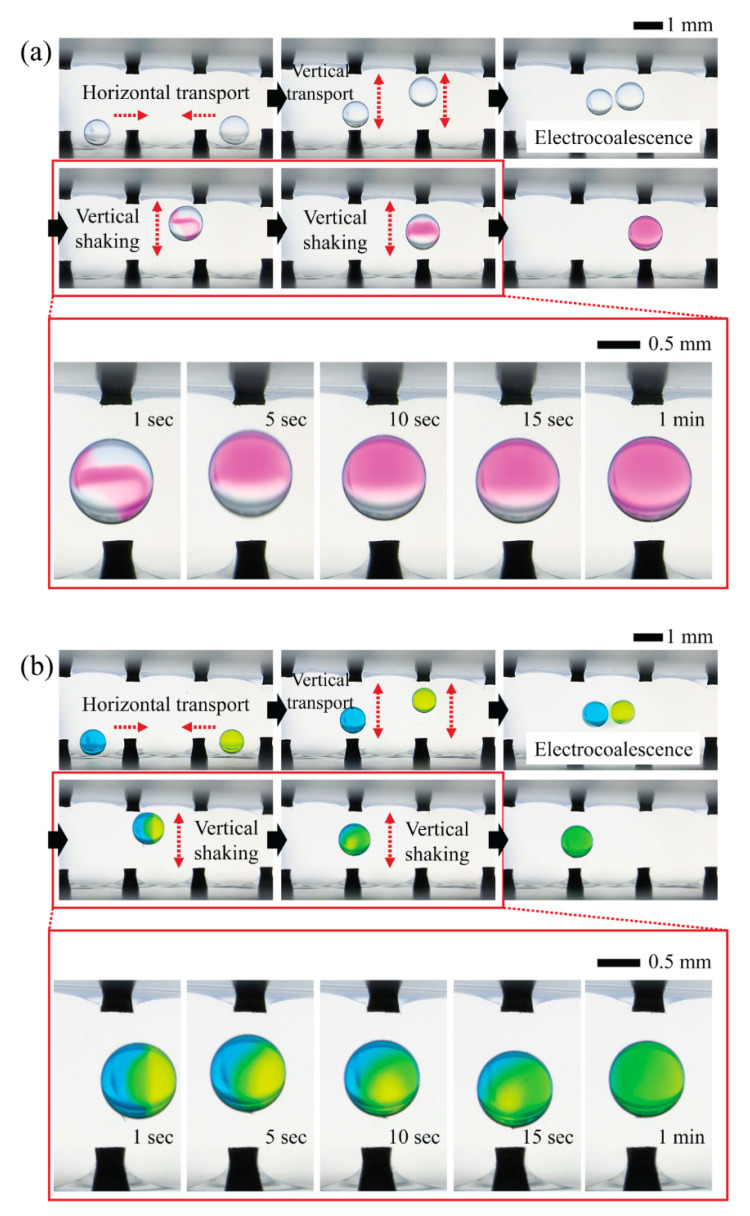
Programmable droplet handling procedure, including horizontal and vertical transport of two oppositely charged microdroplets containing different chemicals, merging via electrical interaction, and vertical shaking via vertical reciprocating motion. (**a**) Mixing between a droplet of PHP indicator solution (left) and a droplet of sodium hydroxide aqueous solution (right) (Video S3 in Supplementary Materials). (**b**) Mixing between a blue dye droplet (left) and a yellow dye droplet (right) (Video S4 in Supplementary Materials). The distance between the pin electrodes is 2 mm in this case, and the applied voltage is 400 V.

We also demonstrate the feasibility of programmable 3D droplet handling by horizontally and vertically transporting two oppositely charged microdroplets containing different chemicals, merging them, and shaking them vertically via vertical reciprocating motion (Figure 5). The following is the detailed droplet handling procedure. First, at both ends, two droplets of PHP indicator solution and NaOH solution come in contact with the positive electrode and charge with a positive charge. Second, the droplets can be moved horizontally toward each other by sequentially applying a ground signal to neighboring electrodes. Third, the droplets can be moved vertically by applying a ground signal to the upper electrode. Fourth, the droplets vertically reciprocate between the upper and lower electrodes and take on opposite charges from a certain point due to the difference in vertical reciprocation speed caused by the density difference between the two droplets. The densities of the NaOH aqueous solution and the PHP indicator solution, in this case, are 1.01 g/cm^3^ and 0.78 g/cm^3^, respectively. Fifth, the droplets with opposite charges electrically merge when they touch. Finally, the coalesced droplets reciprocate vertically between the lower and upper electrodes, completing the mixing process within roughly one minute. A quantitative analysis is required to determine which of the horizontal reciprocating and vertical reciprocating motions has the greater mixing efficiency, which will be described and discussed later. Similarly, we demonstrate that droplets of different colors can be three-dimensionally transported, merged, and mixed via vertical reciprocation motion using the same droplet handling procedures, as shown in Figure 5b.

**Figure 6:**
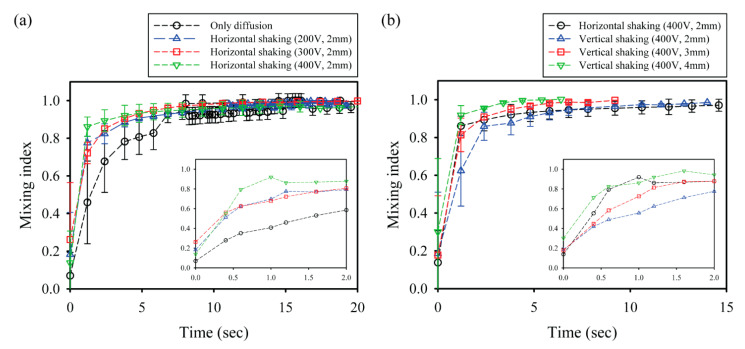
(**a**) Temporal variation in the mixing index as a function of applied voltage in the case of horizontal shaking via horizontal reciprocating motion. (**b**) Temporal variation in the mixing index as a function of electrode distance in the case of vertical shaking via vertical reciprocating motion. The insets of the graphs exhibit temporal variation in the mixing index at the early stage (within nearly 2 s). The graph insets show temporal variation in the mixing index at an early stage (within nearly 2 s).

To conduct quantitative analysis and comparison of the mixing performance of CCEP-actuated shaking droplets, the temporal variation in the mixing index is acquired using digital image processing and data analysis with an in-house MATLAB-based tool, as illustrated in Figure 6. First, the effect of voltage on the mixing index within a shaking droplet via horizontal reciprocating motion driven by CCEP actuations at different voltages is investigated. The pure diffusion experiment is carried out in the current work by manually and gently merging two droplets of distinct reagents through a micropipette tip. Regardless of the applied voltage, all the droplets exhibit the steepest increase up to mixing index values ranging from 0.7 to 0.8 at the early stage (within nearly 2 s), after which they gradually increase with a gentle curve, as shown in Figure 6a. As the applied voltage increases, the slope of the steeply growing portion mildly increases and the time necessary for complete mixing slightly decreases. This may be because the frequency of horizontal reciprocating motion intensifies as the applied voltage increases. Here, the average frequencies of horizontal reciprocating motions at applied voltages of 200 V, 300 V, and 400 V are measured to be approximately 2.6 Hz, 4.7 Hz, and 7.4 Hz, respectively. 

We also evaluate the influence of the distance between the lower and upper electrodes on the mixing index within a shaking droplet subjected to vertical reciprocating motion caused by the CCEP actuation, as shown in Figure 6b. Because the applied voltage is constant, this is the same as examining at the influence of the electric field. The growing tendency of the overall mixing index is confirmed to begin abruptly and then steadily increase, comparable to the horizontal reciprocating motion. Interestingly, even though the strength of the electric field decreases as the distance between the electrodes increases, the mixing time (i.e., the time required to achieve a mixing index of 0.95) is confirmed to be shorter. This may be because when the electrode distance is small, vertical reciprocating motion occurs at an extremely short distance for inertial force caused by gravity to develop; however, when the electrode distance is increased, the inertial force can positively affect the mixing. In this study, total mixing in the shortest time (approximately 2.4 s) can be achieved by vertical reciprocation motion at experimental conditions with an electrode distance of 4 mm and an applied voltage of 400 V.

**Figure 7:**
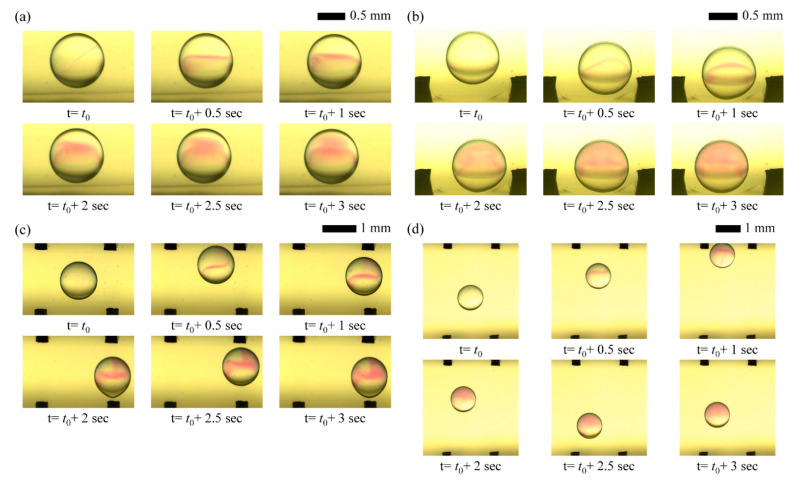
High-speed images of mixing flow patterns driven by (**a**) only diffusion and (**b**–**d**) reciprocation motions: (**b**) horizontal reciprocation motion with an electrode distance of 2 mm and an applied voltage of 400 V; (**c**) vertical reciprocation motion with an electrode distance of 2 mm and an applied voltage of 400 V; (**d**) vertical reciprocation motion with an electrode distance of 4 mm and an applied voltage of 400 V.

We succeed in visualizing mixing flow patterns within droplets driven by only diffusion and reciprocation motions under different CCEP actuation conditions by a high-speed imaging system, as shown in Figure 7. Without an external stimulus, mixing proceeds slowly via diffusion from NaOH to the PHP indicator solution within a stationary droplet, as shown in Figure 7a. When the droplet is shaken horizontally using CCEP actuation, the internal convection flows slightly facilitate the mixing process (Figure 7b). It can be visually confirmed that the mixing process gets slow when the droplet is shaken vertically at the same electrode spacing and applied voltage conditions as in the horizontal shaking situation since the inertial force owing to gravity does not develop well, shown in Figure 7c. However, if the moving distance where the inertial force is developed is secured, it can be confirmed that mixing occurs rapidly due to internal convection flow. However, as the distance between the electrodes increases and inertia develops during the droplet’s vertical reciprocating motion, it is demonstrated that fast mixing is achievable due to internal convection flow (Figure 7d).

## 4. Conclusions

We created a 3D DMF platform based on CCEP using two parallel electrode modules constructed from ordinary pin header sockets and a 3D-printed housing. We successfully demonstrated the feasibility of programmable 3D droplet handling on the platform, including horizontal and vertical transport of two oppositely charged microdroplets containing distinct chemicals, electrocoalescence, and mixing via horizontal and vertical reciprocating motions of the droplets. In addition, the mixing performance of CCEP-actuated shaking droplets was systematically assessed using quantitative analysis of the temporal chromic change caused by the chemical reaction between the pH indicator and base solutions within the shaking droplets. Finally, mixing flow patterns within the shaking droplets were visualized by a high-speed imaging system, which can indirectly elucidate differences in mixing performance under various CCEP actuation situations. To actually adopt the 3D DFM platform proposed in this study to biochemical and biomedical applications, multi-step droplet handling operations such as droplet formation, coalescence, splitting, and transporting will be required. As a result, to extend the CCEP-based DMF function, we will preferentially integrate an EHD jetting-based droplet production system [22] and a CCEP-based 3D droplet manipulation system in the near future. While the droplet handling function requires further advancement, The proposed CCEP-steerable 3D droplet manipulation and active mixing will serve as fundamental and critical strategies to develop 3D DMF platforms for a variety of engineering applications, including biochemical and biomedical applications.

## Data Availability

Not applicable.

## Supplementary Materials

The following supporting information can be downloaded at: https://www.mdpi.com/article/10.3390/mi13040593/s1, Video S1: Horizontal mixing between a droplet of PHP indicator solution and a droplet of sodium hydroxide aqueous solution; Video S2: Horizontal mixing between a blue dye droplet and a yellow dye droplet; Video S3: Vertical mixing between a droplet of PHP indicator solution and a droplet of sodium hydroxide aqueous solution; Video S4: Vertical mixing between a blue dye droplet and a yellow dye droplet.

## References

[1] Fair R.B. (2007). Digital microfluidics: Is a true lab-on-a-chip possible?. Microfluid. Nanofluid..

[2] Choi K., Ng A.H., Fobel R., Wheeler A.R. (2012). Digital microfluidics. Annu. Rev. Anal. Chem..

[3] Jebrail M.J., Bartsch M.S., Patel K.D. (2012). Digital microfluidics: A versatile tool for applications in chemistry, biology and medicine. Lab Chip.

[4] Samiei E., Tabrizian M., Hoorfar M. (2016). A review of digital microfluidics as portable platforms for lab-on a-chip applications. Lab Chip.

[5] Lee J., Moon H., Fowler J., Schoellhammer T., Kim C.J. (2002). Electrowetting and electrowetting-on-dielectric for microscale liquid handling. Sens. Actuator A Phys..

[6] Li J., Kim C.J. (2020). Current commercialization status of electrowetting-on-dielectric (EWOD) digital microfluidics. Lab Chip.

[7] Sourais A.G., Papathanasiou A.G. (2021). Modelling of electrowetting-induced droplet detachment and jumping over topographically micro-structured surfaces. Micromachines.

[8] Wang Z., Liu X., Wang L., Zhao C., Zhou D., Wei J. (2022). Trampolining of droplets on hydrophobic surfaces using electrowetting. Micromachines.

[9] Quilliet C., Berge B. (2001). Electrowetting: A recent outbreak. Curr. Opin. Colloid Interface Sci..

[10] Mugele F., Baret J.C. (2005). Electrowetting: From basics to applications. J. Phys. Condens. Matter.

[11] Mukhopadhyay R. (2005). When microfluidic devices go bad. Anal. Chem..

[12] Im D.J., Yoo B.S., Ahn M.M., Moon D., Kang I.S. (2013). Digital electrophoresis of charged droplets. Anal. Chem..

[13] Samad M.F., Kouzani A.Z., Rahman M.M., Magniez K., Kaynak A. (2015). Design and fabrication of an electrode for low-actuation-voltage electrowetting-on-dielectric devices. Procedia Technol..

[14] Drews A.M., Cartier C.A., Bishop K.J. (2015). Contact charge electrophoresis: Experiment and theory. Langmuir.

[15] Im D.J. (2015). Next generation digital microfluidic technology: Electrophoresis of charged droplets. Korean J. Chem. Eng..

[16] Bishop K.J., Drews A.M., Cartier C.A., Pandey S., Dou Y. (2018). Contact charge electrophoresis: Fundamentals and microfluidic applications. Langmuir.

[17] Jung Y.M., Kang I.S. (2010). Electric charge-mediated coalescence of water droplets for biochemical microreactors. Biomicrofluidics.

[18] Im D.J., Jeong S.N., Yoo B.S., Kim B., Kim D.P., Jeong W.J., Kang I.S. (2015). Digital microfluidic approach for efficient electroporation with high productivity: Transgene expression of microalgae without cell wall removal. Anal. Chem..

[19] Um T., Hong J., Im D.J., Lee S.J., Kang I.S. (2016). Electrically controllable microparticle synthesis and digital microfluidic manipulation by electric-field-induced droplet dispensing into immiscible fluids. Sci. Rep..

[20] Nilsson M.A., Rothstein J.P. (2011). The effect of contact angle hysteresis on droplet coalescence and mixing. J. Colloid Interface Sci..

[21] Lu Y., Zhang M., Zhang H., Huang J., Wang Z., Yun Z., Wang Y., Pang W., Duan X., Zhang H. (2018). On-chip acoustic mixer integration of electro-microfluidics towards in-situ and efficient mixing in droplets. Microfluid. Nanofluid..

[22] Cartier C.A., Graybill J.R., Bishop K.J. (2017). Electric generation and ratcheted transport of contact-charged drops. Phys. Rev. E.

